# Evaluation of Schistosome Promoter Expression for Transgenesis and Genetic Analysis

**DOI:** 10.1371/journal.pone.0098302

**Published:** 2014-05-23

**Authors:** Shuang Liang, Melissa Varrecchia, Kenji Ishida, Emmitt R. Jolly

**Affiliations:** Department of Biology, Case Western Reserve University, Cleveland, Ohio, United States of America; Queensland Institute of Medical Research, Australia

## Abstract

Schistosome worms of the genus *Schistosoma* are the causative agents of schistosomiasis, a devastating parasitic disease affecting more than 240 million people worldwide. Schistosomes have complex life cycles, and have been challenging to manipulate genetically due to the dearth of molecular tools. Although the use of gene overexpression, gene knockouts or knockdowns are straight-forward genetic tools applied in many model systems, gene misexpression and genetic manipulation of schistosome genes *in vivo* has been exceptionally challenging, and plasmid based transfection inducing gene expression is limited. We recently reported the use of polyethyleneimine (PEI) as a simple and effective method for schistosome transfection and gene expression. Here, we use PEI-mediated schistosome plasmid transgenesis to define and compare gene expression profiles from endogenous and nonendogenous promoters in the schistosomula stage of schistosomes that are potentially useful to misexpress (underexpress or overexpress) gene product levels. In addition, we overexpress schistosome genes *in vivo* using a strong promoter and show plasmid-based misregulation of genes in schistosomes, producing a clear and distinct phenotype- death. These data focus on the schistosomula stage, but they foreshadow strong potential for genetic characterization of schistosome molecular pathways, and potential for use in overexpression screens and drug resistance studies in schistosomes using plasmid-based gene expression.

## Introduction

Schistosomes are parasitic worms of the genus *Schistosoma* and are responsible for more than 240 million cases of human schistosomiasis. Schistosomes have complex life cycles and have been challenging to manipulate genetically due to the lack of molecular tools. Although gene knockouts, knockdowns, or gene misexpression are straightforward in many model systems, manipulation of schistosome gene expression has been exceptionally challenging. In addition, plasmid-based transfection inducing gene expression is limited. There is a paucity of approaches to produce stable transgenic schistosome parasites, and schistosome cell lines are currently unavailable. Strategies to investigate and manipulate schistosomes using molecular genetics primarily focus on “loss of function” experiments using RNA interference (RNAi) to inhibit gene function at the post-transcriptional level [1]–[4] and “gain of function” experiments to insert genetic material into schistosomes using plasmid or viral based gene expression [5], [6]. The recent development of the use of polyethyleneimine (PEI) to facilitate transfection of nucleic acids into schistosomes provides a new tool to study transgenesis in the fluke worm [5]. We previously reported on inducing exogenous gene expression by inserting DNA plasmids into schistosome cells using this approach. However, one of the major challenges for the in-depth study of schistosome trangenesis using this approach is the lack of information to select optimal RNA polymerase II (pol II) promoters to drive expression of target genes. Well-characterized promoters are critical to dissect and characterize molecular pathways and for the molecular engineering and manipulation of biological systems. The significance of this is evident in model systems like budding yeast for which a range of well-characterized and regulated promoters have been isolated and developed to produce a series of constitutive promoters with a versatile range of expression capabilities [7]–[13]. Although commercial and endogenous promoters have been reported in the schistosome system [1], [5], [14]–[22], there has been no thorough investigation directly comparing the effectiveness of promoter dynamics in schistosomes. The choice of which promoter to use is usually based on either the convenience or availability of promoters or the desire to test new endogenous promoters. In addition, the commonly accepted expression profile of promoters used in other model organisms is not necessarily directly suitable for the schistosome system, because schistosomes have complex life cycles with distinctive gene expression profiles at each stage [7], [23]–[25].

To address this need, we evaluated and compared the strength of RNA polymerase II promoters for use in schistosome transgenesis using two commercial viral promoters (cytomegalovirus immediate-early (CMV) promoter [20] and simian virus 40 early (SV40 promoter), and four endogenous schistosome promoters, (*S. mansoni* Heat shock protein 70 (SmHsp70) promoter [15], SmActin1 promoter [26]–[28], Sm23 promoter [5], and SmCalcineurin A promoter [17]). CMV and SV40 viral promoters are commonly used in mammalian transgenesis and have relatively high transcriptional outputs across various species [29]. The SmHsp70 and SmActin promoters direct the transcription of proteins that are ubiquitously expressed [15], [27], [30], [31], whereas both Sm23 and SmCalcineurin A generally do so for cell specific proteins [17], [32].

Here, we report the characterization and comparison of promoters for expression in mechanically transformed schistosome schistosomula and monitor expression for several days post transfection. We show that plasmid-based expression is stable for at least seven days post transfection after initial transfection. We also show that plasmid-based gene misexpression can induce schistosome death in a gene-specific manner (overexpression, ectopic expression and misexpression are used synonymously in this report). These investigations provide a basis to select promoters for transgenic studies in schistosomes defined by promoter strength and foreshadow strong potential for genetic overexpression screens and drug resistance studies in parasitic schistosomes using a plasmid-based approach.

## Materials and Methods

### Animals and Parasites

Two Puerto-Rican strains of *Schistosoma mansoni* (NR-21962 and NR-21961), maintained in *Biomphalaria glabrata* snails were obtained from the Biomedical Research Institute (Rockville, MD). Cercariae were shed and transformed into schistosomula as previously described [33], [34]. Transformed schistosomula (∼8,000/well), were maintained in a 12-well cell culture plate (Midsci, St. Louis, MO) with 2 mL of complete Basch Medium (Basch Medium169, 10% fetal bovine serum, 1X Pen/Strep antibiotic) [4], [33] or transfection medium (described below) per well at 37°C and 5% CO_2_. The parasites were incubated for 48 hours or up to 7 days depending on different experiment uses.

### DNA Plasmid Construction

**Figure 1:**
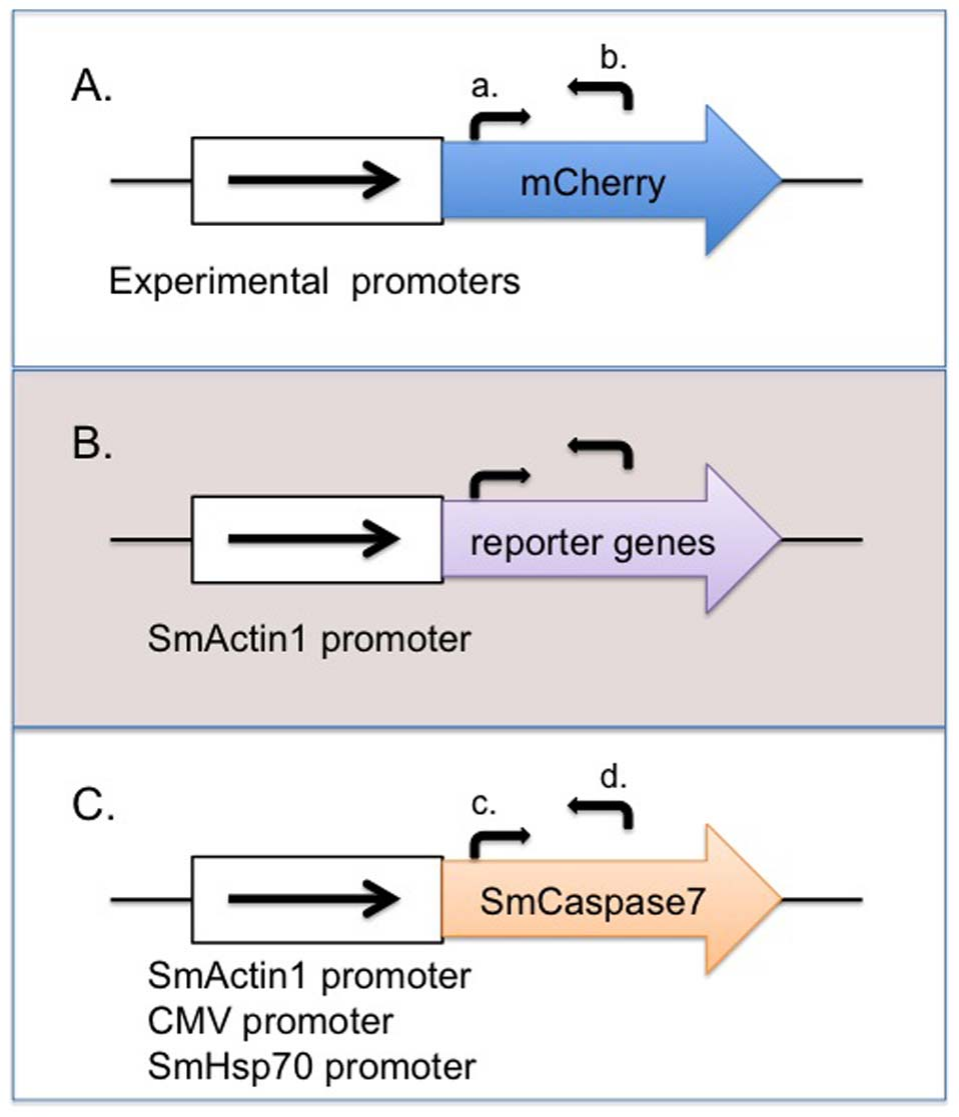
Schematic of expression plasmids used for transfection experiments. (A) Six promoters, two viral (CMV and SV40) and four endogenous (SmHsp70, SmActin1, Sm23, and SmCalcineurinA), were used to regulate expression of an mCherry reporter gene. These promoters were separately subcloned into vector pCI-Neo to make plasmids pEJ1175, pEJ1500, pEJ1501, pEJ1502, pEJ1503, pEJ1504, respectively. Forward arrow (a) and reverse arrow (b) represent forward oligonucleotide (a) and reverse oligonucleotide (b), used to quantify mCherry transcript levels by qRT-PCR. (B) The schistosome Actin1, CyclinB, Caspase3, and Caspase7 genes are separately regulated by the plasmid-based SmActin1 promoter. Forward and reverse arrows represent DNA oligonucleotides used for qRT-PCR analysis of each transcript (Table S2). (C) Transcript levels of the schistosome Caspase 7 gene were regulated by plasmid-based SmActin1, SmHsp70, or CMV promoters. DNA oligos (c) and (d) were used for qRT-PCR analysis to measure SmCaspase7 transcript levels directed by each promoter.

For the initial evaluation of promoters, we amplified all the promoters from either 4 h schistosomula genomic DNA or the pCI-neo plasmid (Promega, Madison, WI) by PCR. The mCherry reporter gene was inserted at the Xbal site of the pCI-neo plasmid (Promega, Madison, WI) to make the vector pEJ1175 as previously described [5]. The cytomegalovirus immediate-early (CMV I.E.) enhancer/promoter region (bp 1–750) of pEJ1175 was then replaced with the following promoters: the 419 bp SV40 promoter cloned from pCI-neo plasmid (bp 2000–2418), the 520 bp promoter of *S. mansoni* Hsp70 (SmHsp70) [15], the 1483 bp promoter of *S. mansoni* Actin 1 (SmActin1) [26], the 1000 bp promoter of *S. mansoni* 23 gene (Sm23) [5], [32], and the1358 bp promoter of *S. mansoni* Calcineurin A (SmCalcineurinA) [17], to make plasmid constructs pEJ1500, pEJ1501, pEJ1502, pEJ1503 and pEJ1504 by In-Fusion HD Cloning (Clontech, Mountainview, CA), respectively. To review, each plasmid was constructed based on the pCI-neo plasmid backbone, containing a distinct RNA pol II promoter, followed by the mCherry reporter gene (Figure 1A) and a SV40 late polyadenylation signal. Primer sequences for PCR amplification of each promoter region and subsequent subcloning into the pCI-neo vector are listed in Table S1.

To assay the viability of schistosomula transfected with plasmids overexpressing different genes, the mCherry reporter gene controlled by the SmActin promoter, was replaced with the *S. mansoni* Actin 1 gene (SmActin1) [35], Cyclin B gene (SmCyclinB) [35], SmCaspase3 gene or SmCaspase7 gene [36], to make plasmid constructs pEJ1505, pEJ1506, pEJ1507 and pEJ1508, respectively. The SmCaspase7 gene replaced the mCherry reporter in plasmids pEJ1501 and pEJ1175, to make plasmid constructs pEJ1509 and pEJ1510, respectively. To review, plasmids pEJ1505, pEJ1506, pEJ1507 and pEJ1508 were constructed with different reporter genes (SmActin1, SmCyclinB, SmCaspase3 or SmCaspase7, respectively) downstream of the SmActin1 promoter (Figure 1B). The SmCaspase7 reporter in pEJ1509 and pEJ1510 is under control of the SmHsp70 or the CMV promoter, respectively (Figure 1C).

### PEI-mediated Transfection

Polyethyleneimine mediated transfection was carried out as previously described [5]. Briefly, each DNA plasmid (4.8 µg) and PEI reagent (7.2 µL of 1 mg/mL) were thoroughly mixed in a total of 2 mL complete Basch Medium to make a transfection medium with a PEI nitrogen to DNA phosphate (N/P) ratio of 11∶1. The transfection mixture was incubated at 37°C for 30 min, then added to either ∼8,000 schistosomula per well in a 12-well culture plate or ∼200 schistosomula per well in a 24-well culture plate. For RNA/protein extraction and caspase activity experiments, 2 mL of transfection mixture were added to ∼8,000 schistosomula per well in a 12-well culture plate and incubated for 48 h at 37°C and 5% CO_2_. For viability experiments, 2 mL of transfection mixture were added to ∼200 schistosomula per well in a 24-well culture plate and cultured for 7 days. The transfection mixture was changed every other day. To assess schistosome viability using propidium iodide treatment over a 7-day period, the sample medium was changed every other day, but no new PEI reagent or DNA plasmid was added after the initial 2-day transfection period. In addition, RNA was extracted every other day to assess mCherry expression level by qRT-PCR. Gene accession numbers for each gene are identified in Table S2.

### RNA Extraction

After 48 h incubation in transfection media, schistosomula were harvested and rinsed twice with 1.5 mL of PBS. Schistosomula were subsequently treated with 50 U of DNAse I (NEB, Ipswich, MA) to remove any external DNA plasmid contamination. Total RNA was extracted according to the PureLink RNA Mini Kit TriZol reagent protocol (Invitrogen, Carlsbad, CA), including an on-column DNAse I digestion. RNA concentration and quality were evaluated on a Nanodrop 8000 spectrophotometer (Thermo Scientific, Waltham, MA).

### Gene Transcript Analysis

Total RNA (∼600 ng) from each transfected sample was converted to cDNA using the First Strand cDNA Synthesis Kit (Invitrogen, Carlsbad, CA). qRT-PCR reactions were carried out using Power SYBR Green Master Mix (Applied Biosystems, Foster City, CA) and the endogenous cyclophilin gene as an internal control, as previously described [5]. To rule out any possible DNA contamination, no-reverse-transcriptase controls were carried out in parallel with experimental samples using equal amounts of RNA from transfected samples. The qPCR was performed on a StepOnePlus Real-Time PCR system (Applied Biosystems, Foster City, CA) programmed for 42 cycles of the following temperature schedule: 94°C 15 sec, 60°C 30 sec, 72°C 30 sec. Detection of the SYBR Green fluorescent intensity occurred at 72°C of each cycle and was analyzed by StepOne System software (Applied Biosystems, Foster City, CA) using ΔΔC_t_ method. qRT-PCR data were verified by t-test, and a p-value less than 0.05 was set as a statistically significant criterion [37].

To determine the transcript levels of the mCherry reporter gene, regulated by the various promoters listed above, primers (forward oligonucleotide oLS219 and reverse oligonucleotide oLS220, Table S2) were designed to amplify a 246 bp mCherry gene fragment by quantitative RT-PCR (qRT-PCR) (Figure 1). To compare the ΔΔC_t_ level of exogenous mCherry gene expression among different transfected samples, the mCherry gene under control of the CMV promoter (pCMV:mCherry) was normalized as the control group (The comparative expression level of pCMV:mCherry was set to one). Equal amounts of cDNA from parasite samples without transfection were used as negative controls to rule out any possible unspecific amplification.

To determine the expression profile change of the reporter genes, SmActin1, SmCyclinB, SmCaspase3 and SmCaspase7, driven by the SmActin1 promoter in transfected parasites, qRT-PCR was performed to compare target gene expression profiles of transfected schistosomula against the untransfected control. Table S2 lists all sets of primers used for qRT-PCR to detect target gene expression. In addition, cDNA samples from parasites transfected with plasmids pEJ1508, pEJ1509 and pEJ1510 expressing SmCaspase7 were assayed by qRT-PCR, to measure the SmCaspase7 gene transcripts regulated by the SmActin1, SmHsp70, or CMV promoters, respectively. All qRT-PCR reactions used the schistosome cyclophilin gene as an endogenous control. The experimental conditions and data analysis were performed as described above.

### Protein Extraction and Western Blot Analysis

To analyze protein expression of the mCherry reporter gene under control of the SV40 promoter, a western blot analysis was performed. First, ∼8,000 schistosomula were transfected (as described above) with plasmid pEJ1500. Schistosomula transfected with pEJ1175 (the CMV promoter driving mCherry reporter) were used as a positive control and untransfected schistosomula as a negative control. Total protein extraction was carried out as previously described [5]. Briefly, 48h post-transfection samples were collected and lysed. Then, 0.5 µg of total protein extract was loaded and separated on a NuPAGE 4–12% Bis-Tris ready-made gel (Invitrogen, CA). The separated protein was transferred to a nitrocellulose membrane (Thermo Scientific, MA) and blocked in PBS with 5% milk and 0.1% Tween-20. The mCherry protein was detected by incubation with 1∶500 mouse anti-mCherry monoclonal primary antibody (Novus Biologicals, Littleton, CO) and 1∶5000 secondary goat anti-mouse IgG-HRP antibody (Santa Cruz Biotechnology, CA). Chemilluminescence was detected and captured with a CCD camera.

### Schistosomula Viability Measurement

Approximately 200 newly transformed schistosomula in three biological replicates were incubated with 2 mL/well of transfection media (as described above) in a 24-well culture plate at 37°C and 5% CO_2_ for 7 days. Parasites used for the viability study were transfected with plasmids carrying the mCherry, SmActin1, SmCyclinB, SmCaspase3 or SmCaspase7 genes driven by the SmActin1 promoter (pEJ1502, pEJ1505, pEJ1506, pEJ1507 and pEJ1508, respectively), or the SmCaspase7 gene driven by the SmHsp70 or CMV promoters (pEJ1509 and pEJ1510, respectively). Schistosomula incubated in the absence of plasmids (PEI Control) and in the absence of both PEI and plasmids (Wild-Type Control) were used as negative controls. The transfection medium was changed every two days. Every 24 hours, survivability rates of transfected schistosomula were determined by observing and manually counting live schistosomula under a light microscope. Parasites displaying a complete loss of motility or a distinct loss of morphological integrity were counted as dead. Schistosomula viability was also assessed using propodium iodide staining. We collected 7-day schistosomula transfected with plasmid constructs expressing the SmCaspase7 gene or mCherry gene under control of the SmActin 1 promoter, and schistosomula incubated with PEI alone but without DNA plasmid, rinsed them twice with PBS and stained with 2 µg/mL propidium iodide at 37°C for 15 min. The schistosomula were observed under a microscope with either polarized light or a rhodamine filter (536 nm) under 40X magnification. The dead parasites were visualized under the rhodamine filter.

### Caspase Activity Assay

To quantify the Caspase3/7 activity from parasites expressing SmCaspase3 or SmCaspase7 under control of the SmActin1, SmHasp70, or CMV promoters, caspase protease activity from ∼8,000 transfected schistosomula was assayed using the Caspase-Glo 3/7 kit (Promega, Madison, WI) following the manufacturer's protocol. Briefly, at 48 h post-transfection, schistosomula were harvested, as described above, and resuspended in cell lysis buffer. Samples were then sonicated in six, 15 s pulses (30% amplitude) with 1 min intervals on ice between each pulse [5], [36]. After sonication, equal volumes of cleared cell lysate and Caspase-Glo reagent were mixed and incubated in the dark at room temperature for 1 h. Non-transfected schistosomula were used as a negative control and a no cell lysate sample was used as a background control for luminescence. Caspase activity was measured in an opaque-walled 96-well (Thermo Fisher Scientific, Waltham, MA) plate in a SpectraMax Luminescence Microplate Reader (Molecular Devices, Sunnyvale, CA).

## Results

### Constitutive promoters can induce strong and weak gene expression in schistosomes

**Figure 2:**
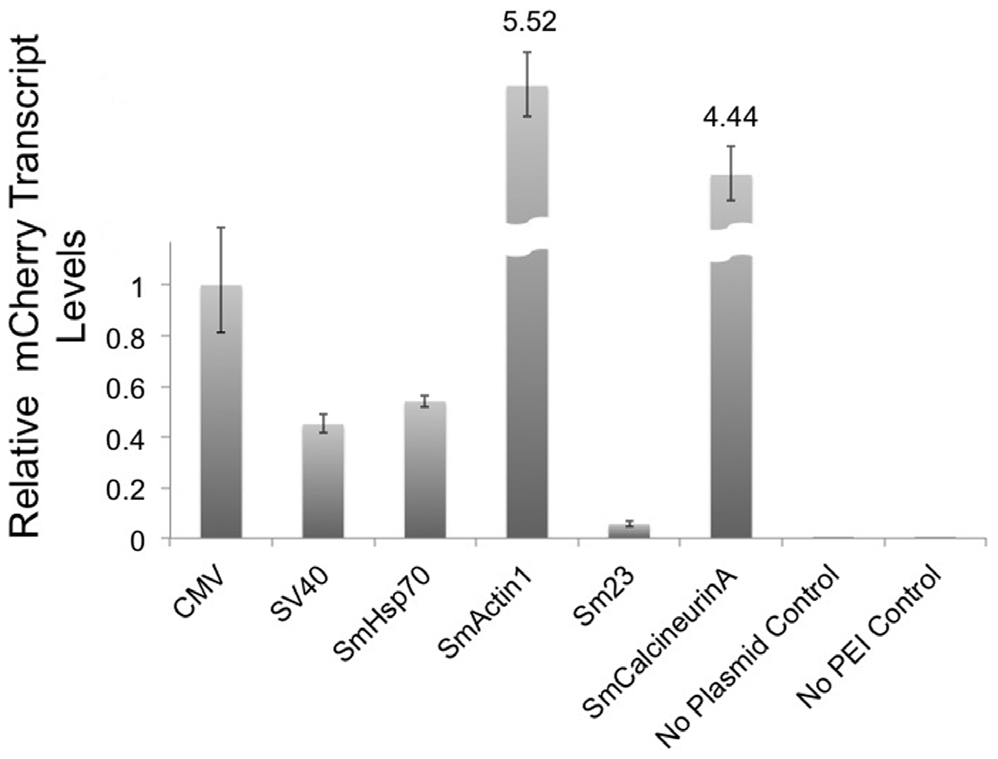
qRT-PCR quantitation of the mCherry transcript in schistosomes transfected with vectors expressing this reporter gene regulated by different promoters. Two viral promoters and four endogenous schistosome promoters were assayed for their ability to express an mCherry reporter. Schistosome samples treated with PEI only or DNA only were used as negative controls. All samples were normalized against the CMV samples.

We assayed the strength of six promoters in schistosome schistosomula by measuring transcript levels of a reporter gene regulated by each promoter using quantitative reverse transcription-PCR (qRT-PCR). Two viral promoters commonly used in mammalian systems, the CMV and SV40 promoters, and four endogenous schistosome promoters, the SmHsp70, SmActin1, Sm23 and SmCalcineurinA promoters, were subcloned into plasmid constructs and transfected into newly transformed schistosomula to assay promoter strength. The mCherry gene was used as a reporter for each of the promoters tested (Figure 1A).Two to seven days after plasmid transfection, total RNA was extracted and DNase I treated, and relative transcript levels of the mCherry reporter genes were measured by qRT-PCR. Both the CMV and SmActin promoters can induce reporter expression in schistosomes [20], [38]. Because the CMV promoter is commonly used in several model systems and is reported as a robust promoter in schistosomes [29], we used the CMV promoter (regulating mCherry transcript- pCMV:mCherry) for normalized comparison to other promoter sequences. We found that SmActin1 and SmCalcineurinA promoters (both endogenous promoters) induce strong transcriptional activity- 5.5-fold and 4.4-fold higher, respectively, than the pCMV:mCherry control (Figure 2). The mCherry transcript levels induced by the SV40 promoter (pSV40:mCherry) and SmHsp70 promoter (pHsp70:mCherry) were approximately 2-fold less than the CMV control (0.54 and 0.45, respectively), indicating that the strength of these two promoters is similar, but less robust than the CMV promoter. mCherry transcript levels induced by the Sm23 promoter (Sm23:mCherry) are only 0.05-fold of the CMV control, much less than any of the other five promoters tested. These data show distinct promoter strength profiles two days after initial transfection in early schistosomula. The SmActin1 and SmCalcineurinA promoters are strong transcriptional activators, and the Sm23 promoter is the weakest at this stage. All data were statistically significant. P-values were less than 0.05 and no signal amplification was detected in the two negative control samples. The negative controls, cDNA from untransfected schistosomula, and a no-RT control, had no signal amplification (data not shown).

**Figure 3:**
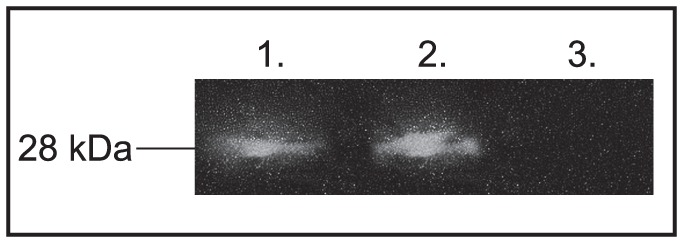
mCherry protein was produced in schistosomula transfected with plasmids expressing mCherry regulated by the SV40 promoter. Total protein was extracted from schistosomula expressing mCherry regulated by the SV40 promoter (Lane 1) and assayed by Western blot analysis using a primary antibody targeting the mCherry protein. The mCherry expression regulated by the CMV promoter was used as a positive control (Lane 2) and total protein from untransfected schistosomula was used as a negative control (Lane 3).

To our knowledge, the full promoter region of SV40 has not been used for schistosome transfection studies. We tested whether the mCherry gene, under control of the SV40 promoter (pSV40:mCherry) could be expressed and translated into protein in schistosomes. Initially we tried to visualize mCherry fluorescence in whole schistosomula, but without success, and so we next tested for mCherry protein expression directly using a primary antibody against the mCherry protein. We identified the 28 kDa mCherry protein signal in pSV40:mCherry transfected schistosome cell extracts by western blot analysis (Figure 3, lane 1). A similar sized band was also detected in the pCMV:mCherry positive control (Figure 3, lane 2), whereas an equivalent protein band was not observed in the untransfected wild-type schistosomula (Figure 3, lane 3). These data emphasize that these promoters can induce transcription and translation of heterologous genes in schistosomes.

### The SmActin1 promoter drives high levels of endogenous gene expression and can induce gene-specific lethality in schistosomes

Since the SmActin1 promoter is a strong activator of reporter gene expression levels, we used this promoter to test whether overexpression of functionally important endogenous genes could disrupt early development in schistosomula and generate a phenotypic change. We cloned the SmActin1 (pActin:Actin), SmCyclinB (pActin:CyclinB), SmCaspase3 (pActin:Caspase3), or SmCaspase7 (pActin:Caspase7) genes so that their expression was regulated by the SmActin1 promoter (Figure 1B), and used these plasmid constructs to transfect newly transformed schistosomula. Each of these genes play important roles in cellular metabolism- Actin is necessary for cellular structural maintenance and motility [30], [39], [40]; Cyclin B proteins are necessary for cell cycle regulation, in particular, exit from mitosis [41]–[43]; and the Caspase genes are involved in cell death and other regulatory functions [44]–[47]. Overexpression of Actin, CyclinB and Caspase genes can result in lethality or damage in animal and yeast models [40], [48]–[55]. Thus, we predicted that if our plasmid-based approach can induce significant overexpression of these genes in schistosomula, then there is strong potential to deleteriously affect schistosome development.

**Figure 4:**
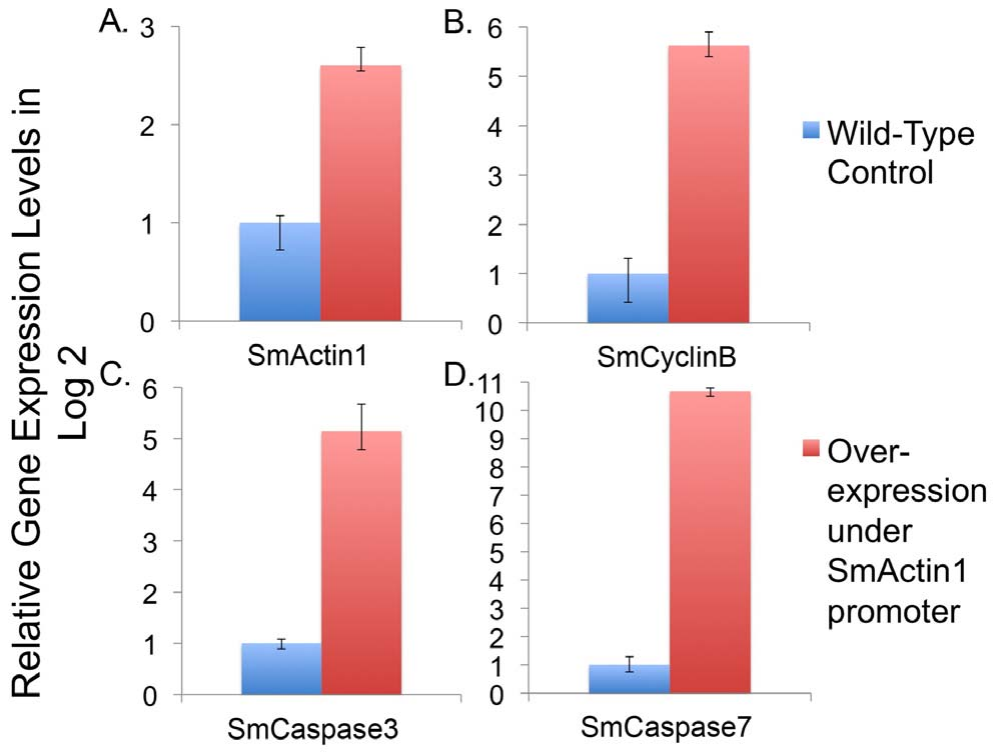
The SmActin1 promoter drives the overexpression of schistosome genes. Four endogenous schistosome genes (SmActin1 (A), SmCyclinB (B), SmCaspase 3 (C), and SmCaspase 7 (D)) were overexpressed from the SmActin1 promoter and compared to untransfected schistosomes (wild-type, negative control). The relative gene transcript levels are shown in log2.

Using qRT-PCR, we compared gene transcript levels between transfected and untransfected schistosomula cultured for 48 hours post-transfection. Transcript levels of all genes were elevated relative to the untransfected, wild-type control (Figure 4). The SmActin1 gene transcript level increased only 3-fold when overexpressed, which is not surprising as it is constitutively expressed during the entire schistosome life cycle [35] (Figure 4A). In contrast, we find that the transcript level of the SmCaspase7 gene in wild-type schistosomula is low (C_T_ value is relatively high compared to other genes, data not shown), so by comparison its expression level was highly elevated (2^10^-fold) in transfected schistosomula. Both SmCyclinB (2^4.6^-fold) and SmCaspase3 (2^4.1^-fold) were similarly upregulated when compared to the control (Figure 4, B and C). The no-RT negative control showed no amplification by qRT-PCR (data not shown). All data were statistically significant (P-values were less than 0.05).

**Figure 5:**
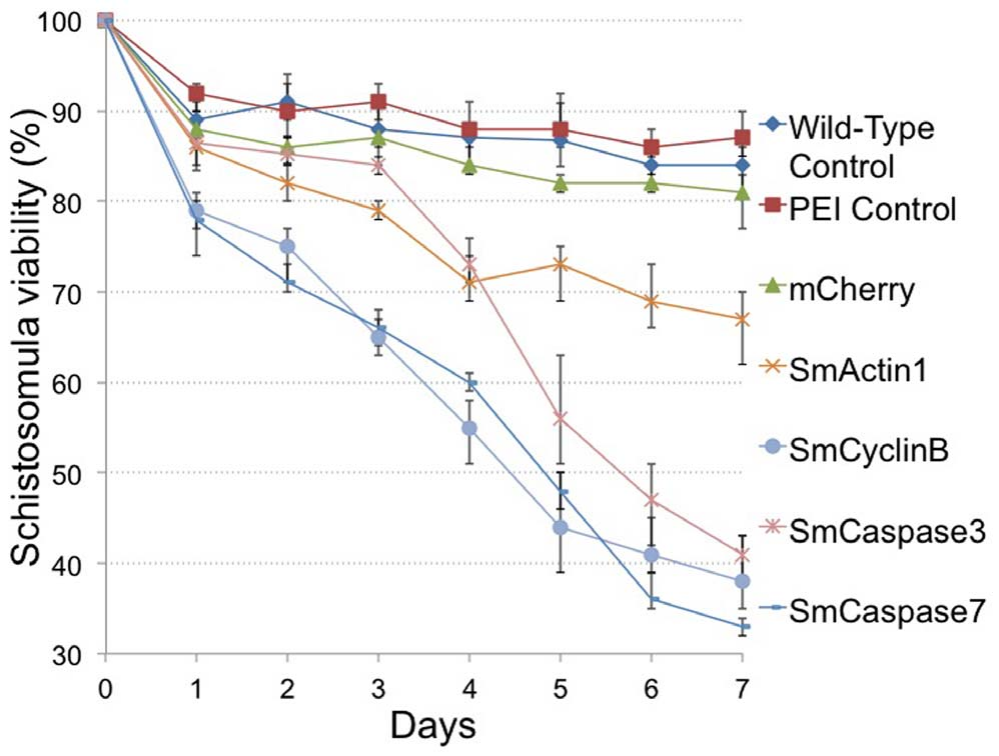
Gene specific overexpression affects schistosome viability. The mCherry gene, SmActin 1 gene, SmCyclin B gene, SmCaspase 3 and 7 genes were cloned under regulation of the SmActin promoter, and expressed in schistosomula for up to 7 days. Viability of schistosomula was assessed and quantified. Samples incubated with neither PEI or plasmid DNA (wildtype) or in the presence of PEI agent alone, were used as experimental controls. Data are shown as the mean percentage of surviving larvae from three biological replicates.

**Figure 6:**
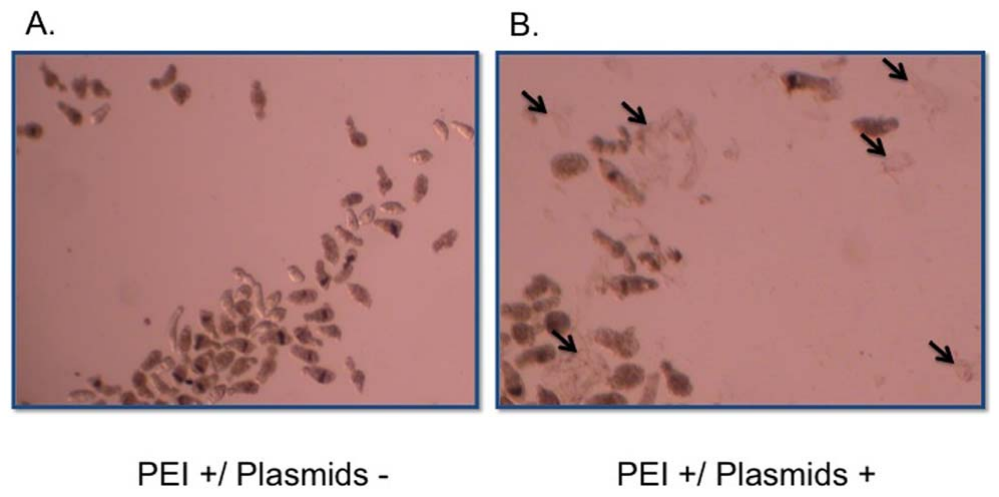
*In vitro* cultured schistosomula in the presence or absence of SmCaspase7 expression plasmids. Parasites were incubated in a total volume of 2°C and 5% CO_2_ for 60 h. Larvae showing dark and opaque in the images may still be alive and are motile when observed under a light microscope. Only parasites displaying a complete loss of motility or a distinctive loss of morphological integrity were considered dead. (A) Schistosomula were cultured in complete Basch medium with PEI agent alone. After 60 h, most parasites were still alive. Similar results were observed with samples treated with DNA constructs alone (data not shown) or samples treated without any transfection agent (wild-type). (B) Schistosomula were incubated with both PEI and plasmid constructs expressing SmCaspase7 under control of the SmActin1 promoter. Overexpression of the SmCaspase 7 gene in schistosomula caused parasite death and lysis as indicated by the large amount of body debris (arrows). Images were obtained under 40X magnification.

Because the SmActin1 promoter has noteworthy increases in target gene expression, we addressed whether these increases in transcript level could produced a change in phenotype. To test this, we incubated ∼200 newly transformed schistosomula in transfection media for 7 days in triplicate. Every 24 hours, live schistosomula were counted (Figure 5). Only parasites displaying a complete loss of motility or a distinct loss of morphological integrity were considered dead. Figure 6 shows an illustration of debris from dead parasites transfected with plasmids expressing SmCaspase7 at 60 h post-transfection. A majority of parasites in the control group remained intact and motile. Remarkable high mortality rates were observed in transfected schistosomula overexpressing SmCaspase3, SmCaspase7, and the SmCyclinB genes when observed daily for 7 days. Almost 70% of schistosomula were dead after overexpression of SmCaspase7, and 62% and 59% of schistosomula were dead when overexpressing SmCyclinB and SmCaspase3, respectively (Figure 5). Overexpression of Actin was not as lethal, and after 7 days the schistosomula had 33% lethality. (Figure 5). In contrast, the negative control groups (PEI only control, a control overexpressing mCherry, and unmanipulated schistosomula or wild-type control) had viability rates between 80–90% after 7 days in culture (Figure 5). The high level of viability observed when mCherry is overexpressed is similar to untransfected schistosomula and indicates that the lethality observed from overexpression of other genes is gene specific and not simply due to random protein overexpression.

### SmActin1, CMV and SmHsp70 promoters induce different levels of elevated SmCaspase7 transcript expression

**Figure 7:**
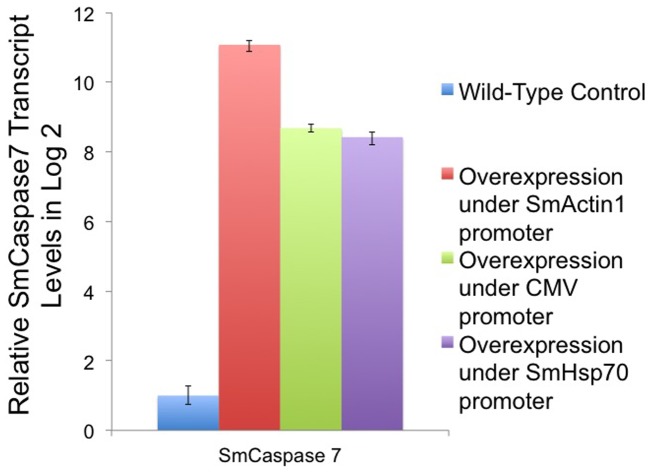
qRT-PCR quantitation of SmCaspase7 transcript in schistosomula expressing plasmid-based SmCaspase7 gene regulated by different promoters. SmCaspase7 was cloned into plasmids and its expression was controlled by the SmActin, CMV, or SmHsp70 promoter. Non-transfected, wild-type schistosomula were used as a negative control. Transcript levels were assessed by quantitative RT-PCR.

Next, we investigated whether the transcript level of an endogenous gene could be differentially regulated from a series of different promoter elements. We introduced plasmids into schistosomula, distinguished by the promoter element, and tested whether schistosome phenotype would vary based on promoter expression level. In the previous experiment, the SmActin1 promoter had the highest transcription efficiency among the six promoters tested (Figure 2). We ranked the activity of the CMV promoter as average, and the SmHsp70 promoter as relatively low at this stage of schistosomula development. We used these promoters to regulate SmCaspase7 gene expression and measured transcript levels (Figure 1C). We assessed the relative SmCaspase7 gene transcript levels from each promoter by qRT-PCR. SmCaspase7 transcripts were significantly elevated under each of the three promoters, Actin (pActin:Caspase7), CMV (pCMV:Caspase7), and SmHeat Shock Protein 70 (pHsp70:Caspase7) (Figure 7), relative to the untransfected control samples. pActin:Caspase7 expression was 4.9-fold (2∧^11-8.7^) higher than SmCaspase7 levels controlled by the CMV promoter. The pHsp70:Caspase7 transcript level was 0.8-fold (2∧^8.4-8.7^) of its expression by the CMV promoter (Figure 7). In comparison, the previous promoter strength experiment showed that the mCherry reporter transcript regulated by the SmActin1 and SmHsp70 promoters were 5.5-fold and 0.56-fold, respectively, relative to mCherry expression level were regulated by the CMV promoter (Figure 2).

**Figure 8:**
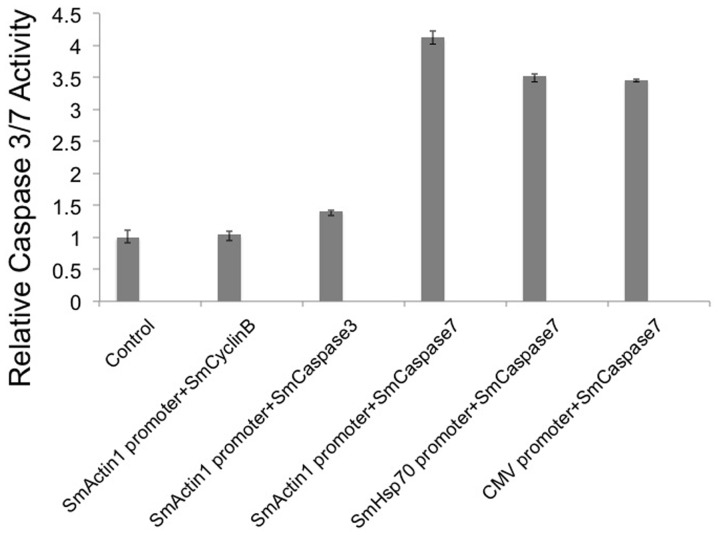
Caspase 3/7 activity in schistosomula is increased by overexpression of SmCaspase7 under different promoters. Caspase 3/7 activity was measured from 8000 schistosomula transfected with plasmids containing either SmCaspase3 or SmCyclinB genes regulated by the SmActin1 promoter, or the SmCaspase7 gene is regulated by three different promoters (SmActin1, SmHsp70, or CMV) and measured after 48 hours. Caspase activity was assayed from cell extracts using the Caspase-Glo 3/7 assay kit (Promega, Madison, WI) following the manufacturer's protocol. Experiments were done in biological triplicates. Untransfected schistosomes are used as a negative control.

To determine whether SmCaspase7 transcript levels from different promoters are consistent with Caspase protein expression, Caspase3/7 enzymatic activity was measured from schistosome samples expressing SmCaspase7 from the SmActin1, SmHsp70 or CMV promoters at 48 hours post transfection (Figure 8; see Materials and Methods). Since high mortality was observed in schistosomula transfected with plasmids overexpressing SmCyclinB and SmCaspase3 from the SmActin1 promoter, we measured the Caspase3/7 activity of these two experimental groups as a control. SmActin should not lead to elevated Caspase3/7 activity served as a good negative control. For overexpression of Caspase3, significant schistosome lethality is not observed until day 4 post transfection, and at two days we observe that SmCaspase3 activity is low (Figure 8). Caspase3/7 activity from a non-transfected control was normalized to one, and lysis buffer alone was used as a background control. No obvious increase in Caspase7 activity, measured by luminescence, was found in schistosomula expressing SmCyclinB, and only slightly elevated (1.4-fold) activity levels was seen in the schistosomula overexpressing SmCaspase3. In contrast, SmCaspase7, induced by the same promoter, showed a 4.1-fold increase of Caspase7 activity. This result is consistent with the viability assay where we observed that Caspase 7 overexpression, but not Caspase 3 overexpression, led to significant schistosomula death two days post transfection (Figure 5). Caspase7 activity in parasites expressing SmCaspase7 induced by either the SmHsp70 promoter or CMV promoter increased 3.5-fold, less than the 4.1-fold increase in activity seen in the sample overexpressing SmCaspase7 under the SmActin1 promoter. Although these results support SmActin1 as the most robust promoter used in these transfection studies, (Figure 2 and Figure 6), it is interesting to note that Caspase7 protease activity increased only slightly in the reporter protein assay.

**Figure 9:**
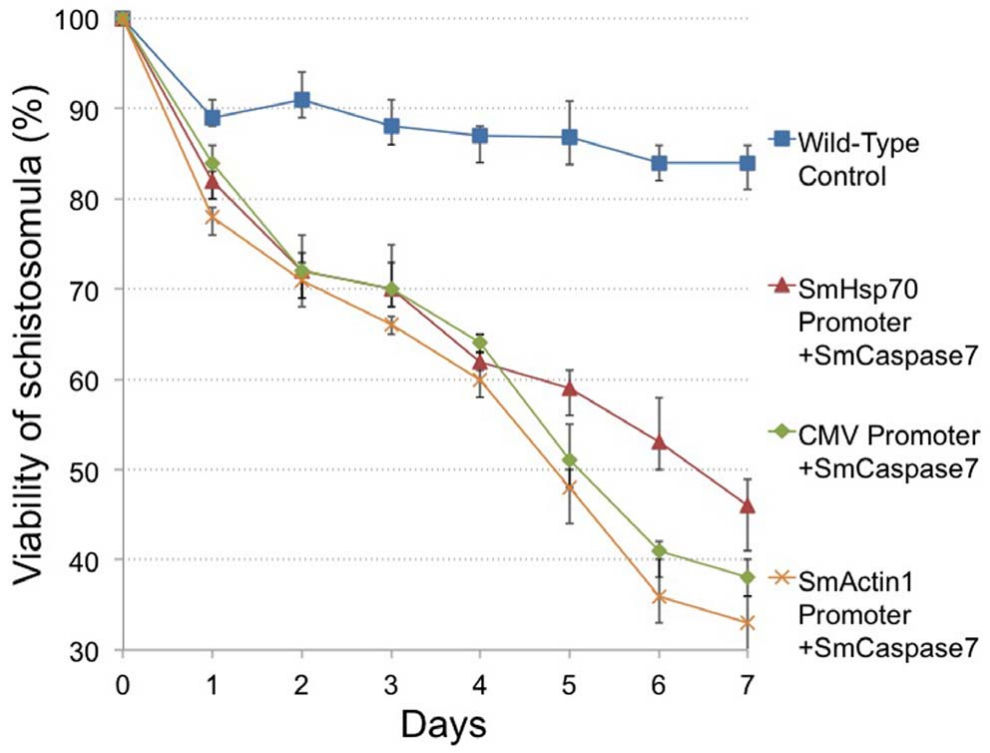
Schistosome viability after SmCaspase7 gene overexpression. Plasmid based Caspase 7 expression was regulated by SmActin1, CMV or SmHsp70 promoters in schistosomula for 7 days. Viability was assessed. Untransfected schistosomula served as an experimental control. Data are shown as a mean percentage of surviving larvae after assessment of three biological replicates.

We next tested whether samples transfected with plasmids carrying SmCaspase7 regulated by either the SmActin1, CMV, or the SmHsp70 promoter had differences in viability over time. (Figure 9). We visually counted triplicates of 200 transfected schistosomula daily for schistosomula viability over a seven day period. After 48 h of incubation, viability of parasites expressing Caspase7 from the SmActin1 promoter, SmHsp70 promoter, or CMV promoter was similar at 71%, 72% and 72%, respectively. After 7 days, viability was reduced to 33%, 46% and 38% for Caspase7 expressed from the SmActin1 promoter, SmHsp70 promoter, or CMV promoters, respectively.

**Figure 10:**
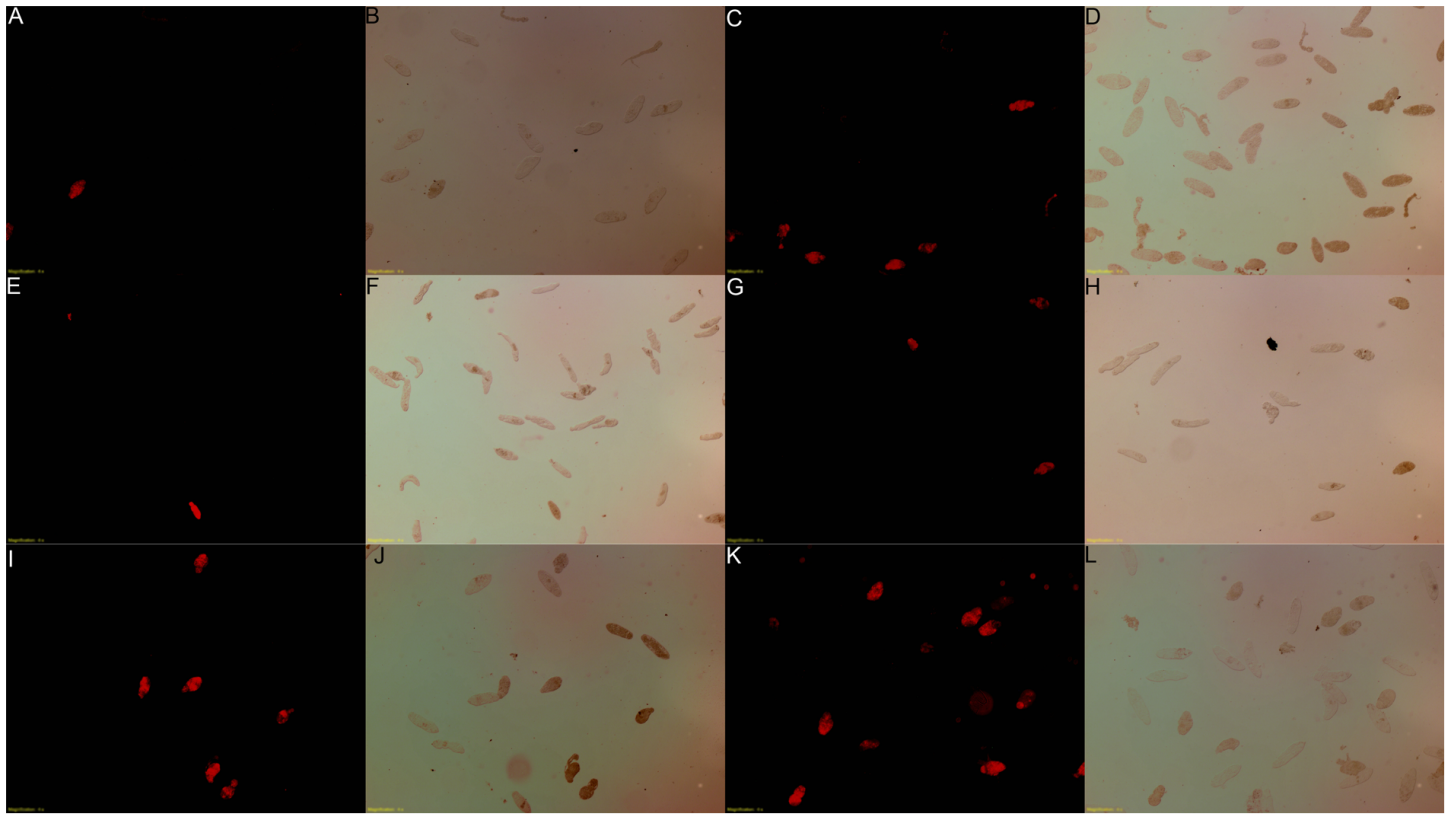
Assessment of 7-day schistosomula viability with propidium iodide. At 7-days, schistosomula were stained with 2 µg/mL propidium iodide and observed under a microscope with either polarized light or a rhodamine filter (536 nm). Dead parasites fluoresce under the rhodamine filter. Schistosomula were cultured in the Basch medium with PEI agent alone (A–D), transfected with plasmid constructs expressing mCherry under control of SmActin1 promoter (E–H), or transfected with plasmid constructs expressing SmCaspase7 under control of SmActin1 promoter (I–L). (A), (C), (E), (G), (I), (K) were detected by a rhodamine filter. (B, D, F, H, I, L) were the corresponding samples visualized by polarized light. Images were obtained under 40X magnification.

To verify our visual analysis of cell viability above, we retested the viability of schistosome parasites using propidium iodide (Figure 10) [56]. Dead schistosomula exposed to propidium iodide fluoresce with a red color when visualized under a rhodamine filter, making quantification of schistosome numbers easier. When we compared schistosomula expressing Caspase from the SmActin promoter (Figure 10, I–L) to the no DNA control (Figure 10, A–D) or pCMV:mCherry (Figure 10, E–H), we found increased cell death due to SmActin overexpresion of SmCaspase7, and these data were similar to our previous observations.

**Figure 11:**
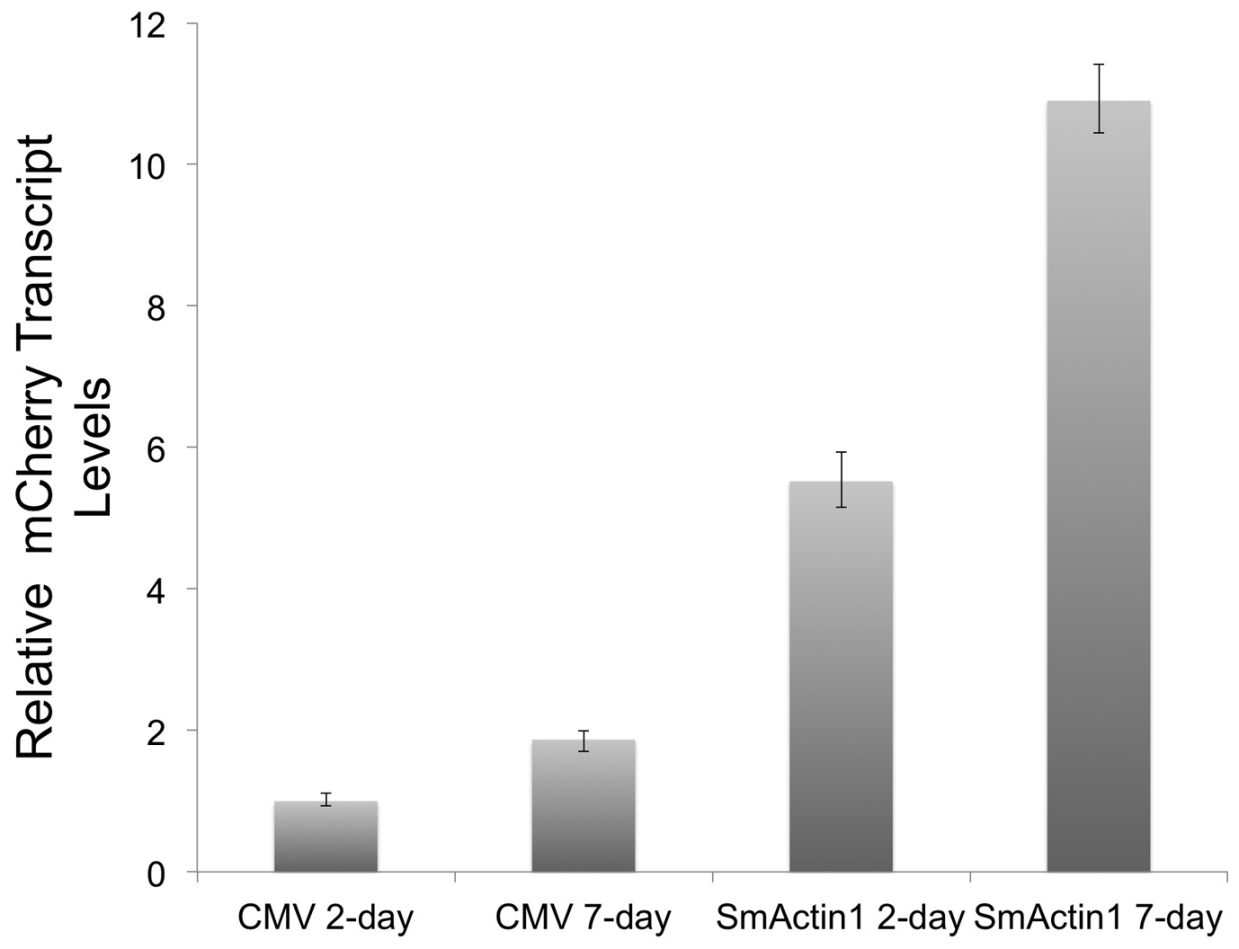
qRT-PCR quantitation of the mCherry transcript in schistosomes transfected with vectors expressing a reporter gene at day 2 and day 7. The viral promoter CMV and the endogenous schistosome promoter SmActin1 were assayed for their ability to express an mCherry reporter at different time frames, without maintaining the schistosomula under transfection conditions.

For the 7-day schistosome tranfections we described earlier, the schistosomula were maintained in transfection media containing PEI and DNA for the entire seven-day period. The media was changed every two days, and after each change new PEI and DNA was added to the original concentration to maintain constant transfection, a significant different between PEI transfection and transfection by electroporation. We addressed whether transfection of pCMV:mCherry and pSmActin:mCherry could induce gene expression over 7 days after an initial transfection but without maintenance in transfection media, in other words, when no new DNA or PEI were added to the schistosomes during media changes. After 7 days, mCherry transcript levels were up 1.8 fold for pCMV:mCherry and 10.9 fold for pSmActin:mCherry relative to pCMV:mCherry at 2 days (Figure 11). The accumulation rate of mCherry transcripts from day 2 to day 7 under the CMV promoter and SmActin1 promoter were similar, at rates of 1.8-fold and 2-fold, respectively. These results indicate that plasmids based gene expression in schistosomula is stable for at least a week post transfection.

## Discussion

Gene expression in biological systems is a complex and tightly regulated process. Thus, the overexpression of genes inappropriately can be disruptive to this delicate balance and result in phenotypic changes (For review [57]). The use of gene overexpression is a powerful tool used in classical genetics to dissect genetic and molecular pathways by identification of gain of function, loss of function and “dominant negative” mutations, and it has been pivotal in particular as it relates to human disease or disease causing agents (for review [57]). The regulation of gene expression levels is an important consideration when designing constructs for genetic analysis, especially in gene therapy [58].

In this report, we characterized commercial and endogenous promoters for expression in schistosomes and identified weak, medium, and strong promoters for use in schistosome transgenesis; and we show that strong overexpression of several genes, SmActin, SmCyclinB, SmCaspase3 and SmCaspase7, can lead to changes in viability in *in vitro* cultured schistosomula. Our analysis testing the strength of endogenous promoters is consistent with gene expression profiles. SmActin1, which participates in cytoskeleton organization, muscle contraction, motility and morphogenesis, is ubiquitously expressed in muscle and tegument cells at all life stages [27], [30], [31]. Supporting this, we found its promoter has the highest transcription levels (Figure 2). Although in schistosomes SmCalcineurinA is normally expressed in tegument cells, excretory tubules and ciliated cells, it functions as a catalytic subunit in a Ca^2+^/calmodulin dependent protein phosphatase, regulating gene expression, ion homeostasis and other cellular processes, and it is expressed throughout the life cycle [17], [59], [60]. On the other hand, SmHsp70 expression is low at the schistosomula stage [61], and the relatively low transcription strength of the SmHsp70 promoter reflects this. With this in mind, a further in-depth analysis to assess the potential of the SmHsp70 promoter expression as an inducible promoter in schistosomes after heat stress may be useful and of interest in future studies. The Sm23 promoter had the lowest level of expression [62]. Sm23 expression normally occurs solely in the tegument, and this may explain the weak transcription outputs of this promoter compared to the other tested promoters. The two viral promoters, CMV and SV40 were both functional in schistosomes albeit at different levels. CMV is a viral promoter widely used across many cell systems [29], and its transcription level in schistosomula is robust. SV40 has not been described in any other schistosome transgenesis study and its expression profile was approximately half that of the CMV promoter (Figures 2). All promoter constructs tested contained the SV40 late polyadenylation signal in the pCI-neo backbone.

The analysis of viability further confirms that the specific PEI transfection agent used in this study does not pose a toxicity problem for *in vitro* cultured schistosomula as previously described [5] (Figure 5, wild-type control). However, strong overexpression of the SmActin1, SmCyclinB, SmCaspase3 or SmCaspase7 genes from the SmActin1 promoter, induced mortality in schistosomula. This correlates with what is generally reported in other systems [40], [48]–[55], [63], although in some cases, gene overexpression causes morphological and developmental changes, as observed in human myoblast cells and esophageal squamous cell carcinoma [39], [40], [42]. The overexpression of each of these genes in schistosomula may alter schistosome early development and be the primary cause of the schistosome lethality observed.

Recently, it was reported that apoptosis occurs in the schistosomula stage with the highest caspase activity at 14 days post-infection to assist in larval development [64]. When we inappropriately expressed SmCaspase3 and SmCaspase7 genes (Figure 5), we found high mortality in transfected schistosomula, likely due to cell apoptosis [36]. This correlates with the effect of caspase overexpression seen in cell culture [65]. However, these data cannot explain how the pro-caspase (zymogens) of SmCaspase3 and SmCaspase7 are activated to induce caspase-dependent apoptosis, nor does it explain why different rates of caspase activity are observed between SmCaspase3 and SmCaspase7 at two days in schistosomula [45], [46].

Our analysis focused on the schistosomula stage of schistosomes. Since gene expression profiles differ between schistosome life cycle stages, we hope to perform a more comprehensive investigation of promoter strengths during other developmental periods as well. It is possible that promoter expression levels can vary depending on the developmental stage as has been reported for the CMV promoter, whose expression can vary by cell type [25], [29], [66], [67]. Correnti et al reported that the “age” of the schistosomula at the time of infection can affect the activity from the SmActin promoter, as measured by a luciferase assay [28]. A further in-depth analysis of stage and “age” dependent plasmid regulation is of keen interest. We are also very interested in identifying the localization of the schistosome transcripts expressed after transgenesis, whether expression is ectopic or localized based on promoter function. It is possible that Sm23 expression will be localized to the tegument although plasmid may exist throughout the schistosomula. Our data also suggest that the protein activity of the reporter gene is not directly proportionate to its transcription levels, strongly suggesting the selection of promoters based on transcription efficiency is not the only factor that determines the resulting phenotype following transgenesis in schistosomes, but that the reporter gene's post-transcriptional and post-translational modifications should also be considered. A general challenge we encountered was the inability to observe mCherry fluorescence in schistosomula, limiting a direct visual observation of transfection. Thus, it was necessary to measure protein expression of mCherry by Western analysis. We hope to resolve this challenge with fluorescent visualization in newly transformed schistosomula.

These investigations represent a more structured approach to genetically assay schistosome biology and increase our potential for schistosome study. The potential for simple transfection of schistosomes combined with a choice in gene expression levels significantly enhances the schistosome genetic toolbox. Low-strength promoters can be used to express genes that are lethal when expressed at high levels. High-strength expression promoters can be used to robustly increase gene expression levels, to purify membrane proteins, or increase membrane or surface exposed proteins for drug-related analysis and resistance studies. Furthermore, the use of overexpression of mutant and wild-type genes will enhance our understanding of basic schistosome biology and provide a useful tool to catalyze schistosome molecular genetics. We predict that these approaches may be applicable to other neglected helminth species.

## Supporting Information

Table S1 Primer sequences used to amplify different promoters of schistosome genes and viral promoter.
(DOCX)

Table S2 Gene names and primer sequences used for quantitative PCR analysis.
(DOCX)

## References

[1] Kalinna BH, Brindley PJ (2007) Manipulating the manipulators: advances in parasitic helminth transgenesis and RNAi. Trends Parasitol 23: 197–204. 17383233 10.1016/j.pt.2007.03.007

[2] Alrefaei YN, Okatcha TI, Skinner DE, Brindley PJ (2011) Progress with schistosome transgenesis. Mem Inst Oswaldo Cruz 106: 785–793. 22124549 10.1590/s0074-02762011000700002 PMC3739710

[3] Skelly PJ, Da'dara A, Harn DA (2003) Suppression of cathepsin B expression in Schistosoma mansoni by RNA interference. Int J Parasitol 33: 363–369. 12705930 10.1016/s0020-7519(03)00030-4

[4] Bhardwaj R, Krautz-Peterson G, Skelly PJ (2011) Using RNA interference in Schistosoma mansoni. Methods Mol Biol 764: 223–239. 21748644 10.1007/978-1-61779-188-8_15

[5] Liang S, Knight M, Jolly ER (2013) Polyethyleneimine Mediated DNA Transfection in Schistosome Parasites and Regulation of the WNT Signaling Pathway by a Dominant-Negative SmMef2. PLoS Negl Trop Dis 7: e2332. 23936566 10.1371/journal.pntd.0002332 PMC3723562

[6] Rinaldi G, Eckert SE, Tsai IJ, Suttiprapa S, Kines KJ, et al (2012) Germline transgenesis and insertional mutagenesis in Schistosoma mansoni mediated by murine leukemia virus. PLoS Pathog 8: e1002820. 22911241 10.1371/journal.ppat.1002820 PMC3406096

[7] Blazeck J, Garg R, Reed B, Alper HS (2012) Controlling promoter strength and regulation in Saccharomyces cerevisiae using synthetic hybrid promoters. Biotechnol Bioeng 109: 2884–2895. 22565375 10.1002/bit.24552

[8] Guarente L (1983) Yeast promoters and lacZ fusions designed to study expression of cloned genes in yeast. Methods Enzymol 101: 181–191. 6310321 10.1016/0076-6879(83)01013-7

[9] Holland JP, Holland MJ (1980) Structural comparison of two nontandemly repeated yeast glyceraldehyde-3-phosphate dehydrogenase genes. J Biol Chem 255: 2596–2605. 6244283

[10] Lu C, Jeffries T (2007) Shuffling of promoters for multiple genes to optimize xylose fermentation in an engineered Saccharomyces cerevisiae strain. Appl Environ Microbiol 73: 6072–6077. 17693563 10.1128/AEM.00955-07 PMC2074996

[11] Reifenberger E, Freidel K, Ciriacy M (1995) Identification of novel HXT genes in Saccharomyces cerevisiae reveals the impact of individual hexose transporters on glycolytic flux. Mol Microbiol 16: 157–167. 7651133 10.1111/j.1365-2958.1995.tb02400.x

[12] Sun J, Shao Z, Zhao H, Nair N, Wen F, et al (2012) Cloning and characterization of a panel of constitutive promoters for applications in pathway engineering in Saccharomyces cerevisiae. Biotechnol Bioeng 109: 2082–2092. 22383307 10.1002/bit.24481

[13] Walfridsson M, Hallborn J, Penttila M, Keranen S, Hahn-Hagerdal B (1995) Xylose-metabolizing Saccharomyces cerevisiae strains overexpressing the TKL1 and TAL1 genes encoding the pentose phosphate pathway enzymes transketolase and transaldolase. Appl Environ Microbiol 61: 4184–4190. 8534086 10.1128/aem.61.12.4184-4190.1995 PMC167730

[14] Davis RE, Parra A, LoVerde PT, Ribeiro E, Glorioso G, et al (1999) Transient expression of DNA and RNA in parasitic helminths by using particle bombardment. Proc Natl Acad Sci U S A 96: 8687–8692. 10411936 10.1073/pnas.96.15.8687 PMC17577

[15] Wippersteg V, Kapp K, Kunz W, Jackstadt WP, Zahner H, et al (2002) HSP70-controlled GFP expression in transiently transformed schistosomes. Mol Biochem Parasitol 120: 141–150. 11849713 10.1016/s0166-6851(01)00446-7

[16] Wippersteg V, Kapp K, Kunz W, Grevelding CG (2002) Characterisation of the cysteine protease ER60 in transgenic Schistosoma mansoni larvae. Int J Parasitol 32: 1219–1224. 12204221 10.1016/s0020-7519(02)00092-9

[17] Rossi A, Wippersteg V, Klinkert MQ, Grevelding CG (2003) Cloning of 5' and 3' flanking regions of the Schistosoma mansoni calcineurin A gene and their characterization in transiently transformed parasites. Mol Biochem Parasitol 130: 133–138. 12946850 10.1016/s0166-6851(03)00158-0

[18] Wippersteg V, Sajid M, Walshe D, Khiem D, Salter JP, et al (2005) Biolistic transformation of Schistosoma mansoni with 5' flanking regions of two peptidase genes promotes tissue-specific expression. Int J Parasitol 35: 583–589. 15862572 10.1016/j.ijpara.2005.02.002

[19] Heyers O, Walduck AK, Brindley PJ, Bleiss W, Lucius R, et al (2003) Schistosoma mansoni miracidia transformed by particle bombardment infect Biomphalaria glabrata snails and develop into transgenic sporocysts. Exp Parasitol 105: 174–178. 14969695 10.1016/j.exppara.2003.11.001

[20] Osman A, Niles EG, Verjovski-Almeida S, LoVerde PT (2006) Schistosoma mansoni TGF-beta receptor II: role in host ligand-induced regulation of a schistosome target gene. PLoS Pathog 2: e54. 16789838 10.1371/journal.ppat.0020054 PMC1479047

[21] Kines KJ, Mann VH, Morales ME, Shelby BD, Kalinna BH, et al (2006) Transduction of Schistosoma mansoni by vesicular stomatitis virus glycoprotein-pseudotyped Moloney murine leukemia retrovirus. Exp Parasitol 112: 209–220. 16530185 10.1016/j.exppara.2006.02.003

[22] Correnti JM, Brindley PJ, Pearce EJ (2005) Long-term suppression of cathepsin B levels by RNA interference retards schistosome growth. Mol Biochem Parasitol 143: 209–215. 16076506 10.1016/j.molbiopara.2005.06.007

[23] Jolly ER, Chin CS, Miller S, Bahgat MM, Lim KC, et al (2007) Gene expression patterns during adaptation of a helminth parasite to different environmental niches. Genome Biol 8: R65. 17456242 10.1186/gb-2007-8-4-r65 PMC1896014

[24] Gobert GN, McInnes R, Moertel L, Nelson C, Jones MK, et al (2006) Transcriptomics tool for the human Schistosoma blood flukes using microarray gene expression profiling. Exp Parasitol 114: 160–172. 16631746 10.1016/j.exppara.2006.03.003

[25] Teschendorf C, Warrington KH Jr, Siemann DW, Muzyczka N (2002) Comparison of the EF-1 alpha and the CMV promoter for engineering stable tumor cell lines using recombinant adeno-associated virus. Anticancer Res 22: 3325–3330. 12530082

[26] Tchoubrieva EB, Ong PC, Pike RN, Brindley PJ, Kalinna BH (2010) Vector-based RNA interference of cathepsin B1 in Schistosoma mansoni. Cell Mol Life Sci 67: 3739–3748. 20339897 10.1007/s00018-010-0345-3 PMC11115793

[27] Beckmann S, Wippersteg V, El-Bahay A, Hirzmann J, Oliveira G, et al (2007) Schistosoma mansoni: germ-line transformation approaches and actin-promoter analysis. Exp Parasitol 117: 292–303. 17531975 10.1016/j.exppara.2007.04.007

[28] Morales ME, Mann VH, Kines KJ, Gobert GN, Fraser MJ Jr, et al (2007) piggyBac transposon mediated transgenesis of the human blood fluke, Schistosoma mansoni. FASEB J 21: 3479–3489. 17586730 10.1096/fj.07-8726com

[29] Qin JY, Zhang L, Clift KL, Hulur I, Xiang AP, et al (2010) Systematic comparison of constitutive promoters and the doxycycline-inducible promoter. PLoS One 5: e10611. 20485554 10.1371/journal.pone.0010611 PMC2868906

[30] Dominguez R, Holmes KC (2011) Actin structure and function. Annu Rev Biophys 40: 169–186. 21314430 10.1146/annurev-biophys-042910-155359 PMC3130349

[31] MacGregor AN, Shore SJ (1990) Immunocytochemistry of cytoskeletal proteins in adult Schistosoma mansoni. Int J Parasitol 20: 279–284. 2358310 10.1016/0020-7519(90)90141-9

[32] Koster B, Strand M (1994) Schistosoma mansoni: Sm23 is a transmembrane protein that also contains a glycosylphosphatidylinositol anchor. Arch Biochem Biophys 310: 108–117. 8161193 10.1006/abbi.1994.1146

[33] Basch PF (1981) Cultivation of Schistosoma mansoni in vitro. I. Establishment of cultures from cercariae and development until pairing. J Parasitol 67: 179–185. 7241277

[34] Milligan JN, Jolly ER (2011) Cercarial transformation and in vitro cultivation of Schistosoma mansoni schistosomules. J Vis Exp. 10.3791/3191 PMC3217644 21876520

[35] Berriman M, Haas BJ, LoVerde PT, Wilson RA, Dillon GP, et al (2009) The genome of the blood fluke Schistosoma mansoni. Nature 460: 352–358. 19606141 10.1038/nature08160 PMC2756445

[36] Dubois F, Caby S, Oger F, Cosseau C, Capron M, et al (2009) Histone deacetylase inhibitors induce apoptosis, histone hyperacetylation and up-regulation of gene transcription in Schistosoma mansoni. Mol Biochem Parasitol 168: 7–15. 19538992 10.1016/j.molbiopara.2009.06.001

[37] Livak KJ ST (2001) Analysis of relative gene expression data using real-time quantitative PCR and the 2(T)(-Delta Delta C) method. Method 25: 402–408. 10.1006/meth.2001.1262 11846609

[38] Correnti JM, Jung E, Freitas TC, Pearce EJ (2007) Transfection of Schistosoma mansoni by electroporation and the description of a new promoter sequence for transgene expression. Int J Parasitol 37: 1107–1115. 17482194 10.1016/j.ijpara.2007.02.011

[39] Lloyd C, Schevzov G, Gunning P (1992) Transfection of nonmuscle beta- and gamma-actin genes into myoblasts elicits different feedback regulatory responses from endogenous actin genes. J Cell Biol 117: 787–797. 1577858 10.1083/jcb.117.4.787 PMC2289461

[40] Peckham M, Miller G, Wells C, Zicha D, Dunn GA (2001) Specific changes to the mechanism of cell locomotion induced by overexpression of beta-actin. J Cell Sci 114: 1367–1377. 11257002 10.1242/jcs.114.7.1367

[41] Brandeis M, Rosewell I, Carrington M, Crompton T, Jacobs MA, et al (1998) Cyclin B2-null mice develop normally and are fertile whereas cyclin B1-null mice die in utero. Proc Natl Acad Sci U S A 95: 4344–4349. 9539739 10.1073/pnas.95.8.4344 PMC22491

[42] Song Y, Zhao C, Dong L, Fu M, Xue L, et al (2008) Overexpression of cyclin B1 in human esophageal squamous cell carcinoma cells induces tumor cell invasive growth and metastasis. Carcinogenesis 29: 307–315. 18048386 10.1093/carcin/bgm269

[43] Zhou XY, Wang X, Hu B, Guan J, Iliakis G, et al (2002) An ATM-independent S-phase checkpoint response involves CHK1 pathway. Cancer Res 62: 1598–1603. 11912127

[44] Alnemri ES, Livingston DJ, Nicholson DW, Salvesen G, Thornberry NA, et al (1996) Human ICE/CED-3 protease nomenclature. Cell 87: 171. 8861900 10.1016/s0092-8674(00)81334-3

[45] Boatright KM, Salvesen GS (2003) Mechanisms of caspase activation. Curr Opin Cell Biol 15: 725–731. 14644197 10.1016/j.ceb.2003.10.009

[46] Kerr LE, McGregor AL, Amet LE, Asada T, Spratt C, et al (2004) Mice overexpressing human caspase 3 appear phenotypically normal but exhibit increased apoptosis and larger lesion volumes in response to transient focal cerebral ischaemia. Cell Death Differ 11: 1102–1111. 15153940 10.1038/sj.cdd.4401449

[47] Lamkanfi M, Kanneganti TD (2010) Caspase-7: a protease involved in apoptosis and inflammation. Int J Biochem Cell Biol 42: 21–24. 19782763 10.1016/j.biocel.2009.09.013 PMC2787741

[48] Chang BD, Broude EV, Fang J, Kalinichenko TV, Abdryashitov R, et al (2000) p21Waf1/Cip1/Sdi1-induced growth arrest is associated with depletion of mitosis-control proteins and leads to abnormal mitosis and endoreduplication in recovering cells. Oncogene 19: 2165–2170. 10815808 10.1038/sj.onc.1203573

[49] Cursio R, Gugenheim J, Ricci JE, Crenesse D, Rostagno P, et al (1999) A caspase inhibitor fully protects rats against lethal normothermic liver ischemia by inhibition of liver apoptosis. FASEB J 13: 253–261. 9973313 10.1096/fasebj.13.2.253

[50] Heald R, McLoughlin M, McKeon F (1993) Human wee1 maintains mitotic timing by protecting the nucleus from cytoplasmically activated Cdc2 kinase. Cell 74: 463–474. 8348613 10.1016/0092-8674(93)80048-j

[51] Jin P, Hardy S, Morgan DO (1998) Nuclear localization of cyclin B1 controls mitotic entry after DNA damage. J Cell Biol 141: 875–885. 9585407 10.1083/jcb.141.4.875 PMC2132764

[52] Liu H, Krizek J, Bretscher A (1992) Construction of a GAL1-regulated yeast cDNA expression library and its application to the identification of genes whose overexpression causes lethality in yeast. Genetics 132: 665–673. 1468625 10.1093/genetics/132.3.665 PMC1205205

[53] Norden C, Liakopoulos D, Barral Y (2004) Dissection of septin actin interactions using actin overexpression in Saccharomyces cerevisiae. Mol Microbiol 53: 469–483. 15228528 10.1111/j.1365-2958.2004.04148.x

[54] Stevenson LF, Kennedy BK, Harlow E (2001) A large-scale overexpression screen in Saccharomyces cerevisiae identifies previously uncharacterized cell cycle genes. Proc Natl Acad Sci U S A 98: 3946–3951. 11274415 10.1073/pnas.051013498 PMC31159

[55] Wang J, Lenardo MJ (2000) Roles of caspases in apoptosis, development, and cytokine maturation revealed by homozygous gene deficiencies. J Cell Sci 113 (Pt 5): 753–757. 10.1242/jcs.113.5.753 10671365

[56] Peak E, Chalmers IW, Hoffmann KF (2010) Development and validation of a quantitative, high-throughput, fluorescent-based bioassay to detect schistosoma viability. PLoS Negl Trop Dis 4: e759. 20668553 10.1371/journal.pntd.0000759 PMC2910722

[57] Prelich G (2012) Gene overexpression: uses, mechanisms, and interpretation. Genetics 190: 841–854. 22419077 10.1534/genetics.111.136911 PMC3296252

[58] Abruzzese RV, MacLaughlin FC, Smith LC, Nordstrom JL (2002) Regulated expression of plasmid-based gene therapies. Methods Mol Med 69: 109–122. 11987771 10.1385/1-59259-141-8:109

[59] Klee CB, Ren H, Wang X (1998) Regulation of the calmodulin-stimulated protein phosphatase, calcineurin. J Biol Chem 273: 13367–13370. 9593662 10.1074/jbc.273.22.13367

[60] Mecozzi B, Rossi A, Lazzaretti P, Kady M, Kaiser S, et al (2000) Molecular cloning of Schistosoma mansoni calcineurin subunits and immunolocalization to the excretory system. Mol Biochem Parasitol 110: 333–343. 11071287 10.1016/s0166-6851(00)00287-5

[61] He S, Yang L, Lv Z, Hu W, Cao J, et al (2010) Molecular and functional characterization of a mortalin-like protein from Schistosoma japonicum (SjMLP/hsp70) as a member of the HSP70 family. Parasitol Res 107: 955–966. 20602114 10.1007/s00436-010-1960-5

[62] Lee KW, Shalaby KA, Medhat AM, Shi H, Yang Q, et al (1995) Schistosoma mansoni: characterization of the gene encoding Sm23, an integral membrane protein. Exp Parasitol 80: 155–158. 7821405 10.1006/expr.1995.1018

[63] Gomez LA, de Las Pozas A, Reiner T, Burnstein K, Perez-Stable C (2007) Increased expression of cyclin B1 sensitizes prostate cancer cells to apoptosis induced by chemotherapy. Mol Cancer Ther 6: 1534–1543. 17513602 10.1158/1535-7163.MCT-06-0727

[64] Han H, Peng J, Gobert GN, Hong Y, Zhang M, et al (2013) Apoptosis phenomenon in the schistosomulum and adult worm life cycle stages of Schistosoma japonicum. Parasitol Int 62: 100–108. 23159324 10.1016/j.parint.2012.09.008

[65] Marcelli M, Shao TC, Li X, Yin H, Marani M, et al (2000) Induction of apoptosis in BPH stromal cells by adenoviral-mediated overexpression of caspase-7. J Urol 164: 518–525. 10893637

[66] Brooks AR, Harkins RN, Wang P, Qian HS, Liu P, et al (2004) Transcriptional silencing is associated with extensive methylation of the CMV promoter following adenoviral gene delivery to muscle. J Gene Med 6: 395–404. 15079814 10.1002/jgm.516

[67] Meilinger D, Fellinger K, Bultmann S, Rothbauer U, Bonapace IM, et al (2009) Np95 interacts with de novo DNA methyltransferases, Dnmt3a and Dnmt3b, and mediates epigenetic silencing of the viral CMV promoter in embryonic stem cells. EMBO Rep 10: 1259–1264. 19798101 10.1038/embor.2009.201 PMC2756565

