# Correction: Evaluation of Schistosome Promoter Expression for Transgenesis and Genetic Analysis

**DOI:** 10.1371/journal.pone.0212691

**Published:** 2019-02-15

**Authors:** Shuang Liang, Melissa Varrecchia, Kenji Ishida, Emmitt R. Jolly

After publication of this article [1], concerns were raised about Fig 3. There are similarities in background below the bands in lanes 1 and 2. The authors explain that the original image contained streaks in lane 2 caused by Saran wrap used to cover the membrane. The authors attempted to remove these streaks, by copying and pasting a clear portion of the background. The authors confirm that no modifications were made to the bands in Fig 3.

**Fig 3:**
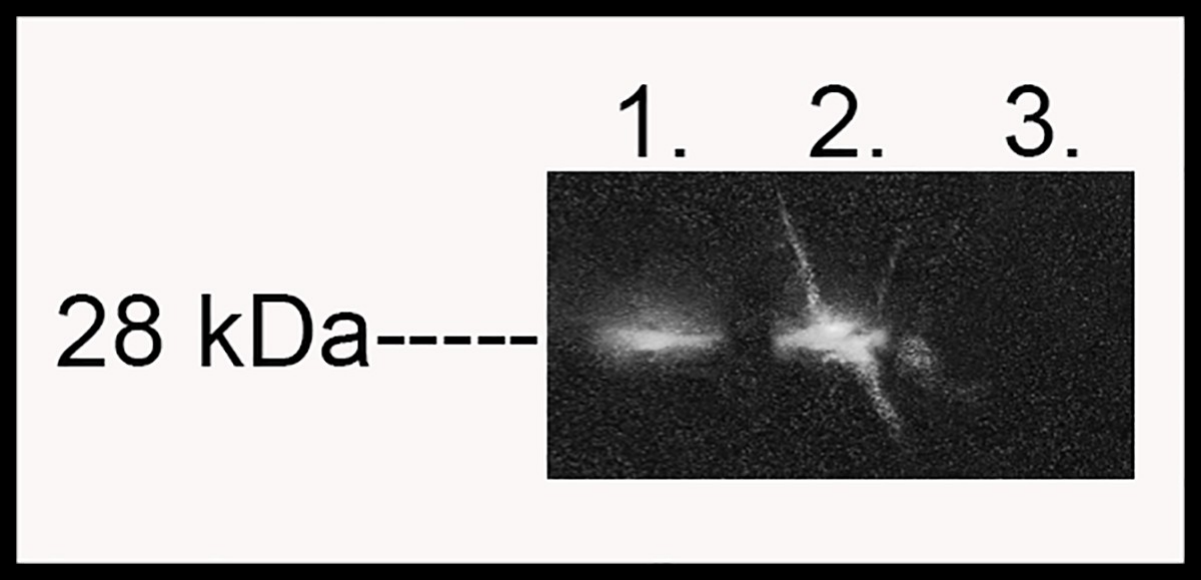
mCherry protein was produced in schistosomula transfected with plasmids expressing mCherry regulated by the SV40 promoter. Total protein was extracted from schistosomula expressing mCherry regulated by the SV40 promoter (Lane 1) and assayed by Western blot analysis using a primary antibody targeting the mCherry protein. The mCherry expression regulated by the CMV promoter was used as a positive control (Lane 2) and total protein from untransfected schistosomula was used as a negative control (Lane 3).

The authors provide a revised version of Fig 3 and the original un-edited and edited blot images as Supporting Information files.

## Supporting information

S1 File Unedited full gel image for Fig 3B containing streaks due to Saran wrap covering the membrane. (TIF)

S2 File Edited full gel image for Fig 3B removing the streaks due to Saran wrap covering the membrane. (TIF)

## References

[1] Liang S, Varrecchia M, Ishida K, Jolly ER (2014) Evaluation of Schistosome Promoter Expression for Transgenesis and Genetic Analysis. PLoS ONE 9(5): e98302 10.1371/journal.pone.0098302 24858918 PMC4032330

