# Supplementary material for: Evaluation of Schistosome Promoter Expression for Transgenesis and Genetic Analysis
Source: PLoS One. 2014 May 23;9(5):e98302. doi: 10.1371/journal.pone.0098302 (PMC4032330; doi:10.1371/journal.pone.0098302)
Supplement: Table S1 — Primer sequences used to amplify different promoters of schistosome genes and viral promoter. (DOCX) [file pone.0098302.s001.docx]

| **Promoter name** | **Length (bp)** | **Forward primer (5’-3’)** | **Reverse primer (5’-3’)** |
| --- | --- | --- | --- |
| SV40 | 419 | GCGCAGCACCATGGCCTGAAATAAC | AAGCTTTTTGCAAAAGCCTAGGCCT |
| SmActin1 | 1483 | TATGGGTAAGCGTTGTTCACAGGAA | TTTACTAGATGATGAGACACTTGAT |
| SmHsp70 | 520 | CACTAAGAAGTAGGCAAACATGACA | TCCAAGATATGTTAAGACGAACAAA |
| Sm23 | 1000 | TGCATGACCGAGCACTGTCAGCTAA | ACTTTCAAACGGGACACAATGCCAA |
| SmCalcineurinA | 1358 | CTGATCTGTGTTGTCAGAGTAGCTA | TAGTGTTGATTAAAATTTTTACTTA |
