# Supplementary material for: Evaluation of Schistosome Promoter Expression for Transgenesis and Genetic Analysis
Source: PLoS One. 2014 May 23;9(5):e98302. doi: 10.1371/journal.pone.0098302 (PMC4032330; doi:10.1371/journal.pone.0098302)
Supplement: Table S2 — Gene names and primer sequences used for quantitative PCR analysis. (DOCX) [file pone.0098302.s002.docx]

| **Primers for qRT-PCR** | | | |
| --- | --- | --- | --- |
| **Gene name** | **Gene ID** | **Forward primer (5’-3’)** | **Reverse primer (5’-3’)** |
| SmCyclophilin | Smp_054330 | TGGGCGGATTTCATAAAGAC | TAAGCATCCAGTGCCAATGA |
| mCherry | HM771696.1 | CCTGTCCCCTCAGTTCATGT | CCCATGGTCTTCTTCTGCAT |
| SmActin1 | Smp_046600 | TGAGCGATTCAGATGTCCAG | CTTCTGCATACGGTCAGCAA |
| SmCyclinB | Smp_082490 | GCGGTTACAAGTTGGACGAT | CTACGCTGGATTTGCCTCTC |
| SmCaspase3 | FJ905905.1 | GTCCTTCCCTGATTGCAAAA | AGAGGAGTTACGCCAAGCAA |
| SmCaspase7 | FJ905906.1 | CTGAGCGTGGACTGTGTGTT | TGTCGGGTTTGAATGCACTA |
