# Supplementary figures and images for: Correction: Evaluation of Schistosome Promoter Expression for Transgenesis and Genetic Analysis
Source: PLoS One. 2019 Feb 15;14(2):e0212691. doi: 10.1371/journal.pone.0212691 (PMC6377129; doi:10.1371/journal.pone.0212691)

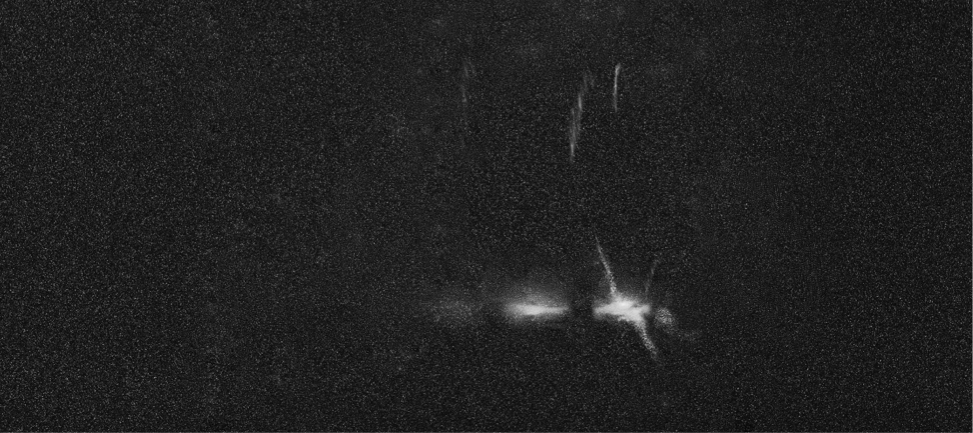

Supplement: S1 File — (TIF) [file pone.0212691.s001.tif]

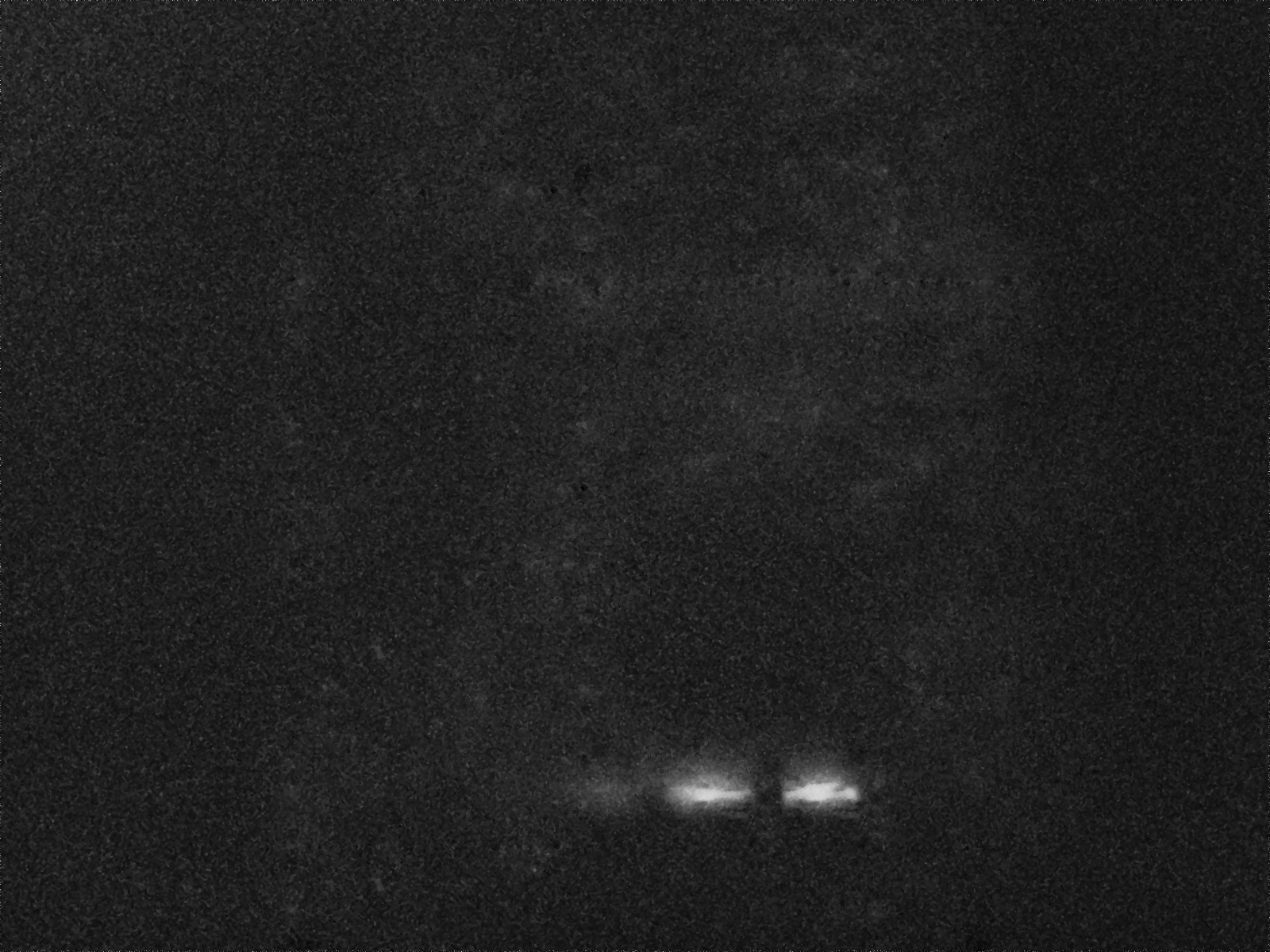

Supplement: S2 File — (TIF) [file pone.0212691.s002.tif]
